# Implications of perceived empathy from spouses during pregnancy for health-related quality of life among pregnant women: a cross-sectional study in Anhui, China

**DOI:** 10.1186/s12884-024-06419-w

**Published:** 2024-04-12

**Authors:** Yu Zhu, Ting Zhu, Hui Wang, Ji-Min Zhu, Dan-dan Zheng, Ping Yin, Bai-Kun Li

**Affiliations:** 1 School of Nursing, Anhui University of Chinese Medicine, Hefei, 230012 China; 2 Pharmacy Department, Suzhou Municipal Hospital, Suzhou, 234099 China; 3 School of Chinese Medicine, Anhui University of Chinese Medicine, Hefei, 230012 China; 4 School of Life Sciences, Anhui University of Chinese Medicine, Hefei, 230012 China; 5 Obstetrical Department, First Affiliated Hospital of Anhui, University of Chinese Medical, Hefei, 230022 China; 6 Nursing Department, Anhui No. 2 Provincial People’s Hospital, Hefei, 230041 China

**Keywords:** Pregnancy, Women, Quality of life, Empathy, Spouse support, Couples

## Abstract

**Background:**

Empathy is a critical component of nursing care, impacting both nurses’ and patients’ outcomes. However, perceived empathy from spouses during pregnancy and its impact on health-related quality of life (HRQoL) are unclear. This study aimed to examine pregnant women’s perceived empathy from their spouses and assess the relation of perceived empathy on HRQoL.

**Methods:**

This cross-sectional study, performed in the obstetric clinics or wards of four well-known hospitals in Anhui Province, China, included 349 pregnant women in the second or third trimester; participants were recruited by convenience sampling and enrolled from October to December 2021. A general information questionnaire, the Interpersonal Reactivity Index (IRI), a purpose-designed empathy questionnaire and the Medical Outcomes Study 12-item Short-Form Health Survey (SF-12) were used to evaluate the pregnant women’s general information, perceptions of empathy and HRQoL. Data were analysed using SPSS 22 at a threshold of *P* < 0.05. Descriptive analysis, Pearson correlation analysis, Student’s *t* test, ANOVA, and multiple regression analysis were used for analysis.

**Results:**

The pregnant women’s total empathy, physical component summary (PCS) and mental component summary (MCS) scores were 41.6 ± 9.0, 41.6 ± 7.6, and 47.7 ± 9.1, respectively. Correlation analysis revealed that the purpose-designed empathy questionnaire items were significantly positively correlated with perspective taking and empathic concern but were not correlated with the personal distress dimension and were only partially correlated with the fantasy dimension. Maternal physical condition during pregnancy, planned pregnancy, and occupational stress were predictors of the PCS score (β = 0.281, *P* < 0.01; β = 0.132, *P* = 0.02; β = -0.128, *P* = 0.02). The behavioural empathy item of our purpose-designed empathy questionnaire and empathic concern were important predictors of the MCS score (β = 0.127, *P* = 0.02; β = 0.158, *P* < 0.01), as well as other demographic and obstetric information, explaining 22.0% of the variance in MCS scores totally (*F* = 12.228, *P* < 0.01).

**Conclusions:**

Pregnant women perceived lower empathy from their spouses and reported lower HRQoL. Perceived empathy, particularly behavioural empathy, may significantly impact pregnant women’s MCS scores but has no effect on their PCS scores. Strategies that foster perceived empathy from spouses among pregnant women are essential for facilitating healthy pregnancies and potentially improving maternal and child health.

## Background

Pregnancy is a very important physiological stage in women of childbearing age. Pregnant women may experience a variety of physiological, psychological, social, and other changes [1], accompanied by varying degrees of stress, anxiety, and depression [2, 3], especially in the second and third trimesters of pregnancy [4]. Moreover, these changes may result in adverse pregnancy outcomes such as preeclampsia and low birth weight, posing serious threats to maternal and infant health and life safety [5, 6].

Societies, especially family members, should pay further attention to pregnant women by providing them with more psychological care and support [3]. The spouse, as the intimate partner of a pregnant woman, is usually the family caregiver most involved in the care of the pregnant woman and can provide practical physical care and emotional support to the pregnant woman. Thus, determining what spouses can do to support and care for pregnant women to improve their HRQoL is a critical and meaningful question.

Health-related quality of life (HRQoL) is considered an important index for the comprehensive evaluation of individual health status and an important nursing and medical outcome [7]; HRQoL is a subjective assessment encompassing physical, mental, and social dimensions [8]. The results of studies on pregnant women’s HRQoL during pregnancy in various countries have been consistent: the HRQoL of pregnant women during pregnancy decreased significantly [7, 9].

Research has identified several factors that influence HRQoL in pregnant women. These include demographic factors such as socioeconomic index scores; higher socioeconomic index scores are positively correlated with HRQOL [10]. Obstetric factors, including being in the third trimester and having high parity, are associated with lower HRQOL scores [10, 11]. Behavioural factors such as physical activity are positively associated with HRQoL [12], while smoking is negatively associated with HRQoL [13]. Adequate sleep quality improves mental HRQOL in the second and third trimesters [7], whereas high perceived stress and depressive symptoms negatively impact HRQOL [13, 14]. Moreover, a 2018 systematic review highlighted factors such as “having family and friends”, “feeling happiness at being pregnant”, and “being optimistic” as contributors to better HRQoL [15]. However, the relationship between pregnant women’s perceived empathy from their spouses during pregnancy and HRQoL has not been explored.

Empathy is defined as a person’s ability to feel the experience of others (emotional empathy), understand these experiences from an objective perspective (cognitive empathy) and express this understanding (behavioural empathy) [16]. Recent research on empathy between spouses is limited, but existing studies suggest that empathy fosters effective communication and strengthens social bonds [17, 18]. A study using the Interpersonal Reactivity Index (IRI) revealed that moderate to high levels of empathy were positively correlated with self-disclosure and relationship intimacy in young breast cancer patients and their spouses [17]. Another study examining 168 infertile couples linked higher IRI scores to improved relationship quality [18].

Previous research, however, has failed to consider pregnant women's perceived empathy from spouses, especially perceptions of the different dimensions of empathy—emotional, cognitive and behavioural empathy—from spouses. Perceived empathy is the degree to which individuals perceive others to empathize with them. To our knowledge, a single Chinese experimental study involving 194 participants demonstrated that accurately perceiving empathy from a romantic partner more effectively alleviates individual social pain and reduces individual recovery time than does perceiving empathy from a friend [19]. Despite its acknowledged importance, the influence of different dimensions of perceived spousal empathy—emotional, cognitive, and behavioural empathy—on HRQoL among pregnant women is presently unclear.

Based on previous studies, we hypothesized that pregnant women’s perceived empathy from spouses is a positive predictor of HRQoL among pregnant Chinese women. This study, therefore, attempted to assess pregnant women’s perceived empathy from spouses and estimate its effect on pregnant women’s HRQoL. The results will be an important source of information on what spouses can do to support their pregnant partners’ health during gestation.

## Methods

### Study design

This was a cross-sectional correlational study. We recruited 349 pregnant women from four hospital settings to participate from October to December 2021. This study was reported according to the Strengthening the Reporting of Observational Studies in Epidemiology (STROBE) checklist [20].

### Participants

Pregnant women were recruited by convenience sampling from the obstetric clinics or obstetric wards of four tertiary hospitals in Anhui Province, China. The inclusion criteria were as follows: (a) married; (b) in the second or third trimester; (c) able to understand the content of the study questionnaire; and (d) able to communicate. Pregnant women who were carrying multiple foetuses or who had comorbid mental disorders were excluded.

### Sample size

This study employed a cross-sectional design, and the sample size was calculated using the G*Power 3.1 program. Referring to a study by Kim and Ko 2018 [21] in which HRQoL was assessed in 203 older Korean adults aged 65 years and older, we calculated that 82 participants would be needed for multiple linear regression analysis along with 30 predictor variables with an effect size of 0.1 or greater, a power of 0.80, and an alpha of 0.05. Considering an assumed 20% attrition rate, a sample 20% greater than the computed value was planned to account for the possibility of incomplete survey data; thus, at least 98 questionnaires needed to be distributed.

### Instruments

The study data were collected using a three-part questionnaire evaluating participants' characteristics, perceived empathy from spouses, and subjective HRQoL.

### Demographic and obstetric information

Pregnant women’s characteristics recorded for this study included demographic items and questions about obstetric features; these items included age, place of residence, education level, occupation, family’s monthly income per capita, height, pre-pregnancy weight, history of smoking and alcohol drinking before pregnancy, exercise before pregnancy, adherence to scientific dietary recommendations, sleep, maternal physical condition during pregnancy, gestational age, gravidity, planned or unplanned nature of the pregnancy, use of assisted reproductive technology, parity, history of adverse pregnancy or childbirth outcomes, and pregnancy complications. Furthermore, occupational stress was measured as a confounding variable using the ‘Occupational stress’ item, which was rated on a 3-point scale (1 = ‘high stress’ to 3 = ‘low or no stress’), and unemployed women were considered to have no occupational stress. All demographic and obstetric information was obtained through self-report questionnaires completed by the participants.

### Pregnant women’s perception of empathy

Pregnant women’s perceived empathy from their spouses was evaluated using the Chinese version of the IRI that measures cognitive and emotional empathy, which was validated by Zhang et al. [22]. This Chinese version of the scale consists of 22 items in four dimensions, namely, perspective taking, fantasy, personal distress, and empathic concern, with good cross-sample consistency and differentiation (*P* < 0.01). Cronbach's α coefficient ranged from 0.532 to 0.758, and test–retest reliability ranged from 0.598 to 0.763. A five-point Likert scale was adopted, with scores ranging from inappropriate to very appropriate (0 ~ 4). The total score ranges from 0 to 88 [22].

We developed a novel empathy questionnaire specifically for assessing nurses' empathy in the context of nursing. The questionnaire comprises six items designed to evaluate the frequency and ability of nurses' emotional, cognitive, and behavioural empathy, with statements such as "I understand the patient/family objectively" [16]. In the present study, we designed a new empathy questionnaire to measure the pregnant women’s perceived empathy from their spouses by performing a comprehensive literature review, referencing the previous empathy questionnaire [16], and consulting with experts. This questionnaire was designed to capture the multidimensional nature of empathy, focusing on emotional, cognitive, and behavioural aspects as they relate to the context of pregnancy. The questionnaire comprises six items designed to evaluate both the frequency and ability of emotional, cognitive, and behavioural empathy. Each item was crafted to evaluate perceived empathy from spouses during pregnancy, with statements such as "During pregnancy, my husband is able to deeply resonate with my feelings,"representing emotional empathy, "During pregnancy, my husband understands me objectively," representing cognitive empathy and "During pregnancy, my husband expresses his understanding to me through verbal or non-verbal communication." representing behavioural empathy. Responses are scored on a 4-point Likert scale, with 1 representing "poor" and 4 representing "very good." This scale was designed to quantify the level of empathy, with higher scores indicating greater empathy. We scored each item separately rather than as an overall score for research purposes. Both the score of frequency and ability of emotional, cognitive, and behavioural empathy range from 1 to 4. The internal consistency of the questionnaire for measuring the frequency and ability of perceived empathy was assessed using Cronbach's alpha, and the results confirmed the questionnaire’s reliability (Cronbach's alpha = 0.846; Cronbach's alpha = 0.893).

### Pregnant women’s HRQoL

Pregnant women’s HRQoL was measured using the Medical Outcomes Study 12-item Short-Form Health Survey (SF-12), the abbreviated form of the Medical Outcomes Study 36-item Short-Form Health Survey (SF-36) [23]. The Chinese version of the SF-12 demonstrated validity and was equivalent to the original English version in terms of the psychometric properties [24]. This universal scale measures HRQoL in the past 4 weeks using 12 items in the following 8 dimensions: general health (GH), physical functioning (PF), role–physical (RP), bodily pain (BP), vitality (VT), social functioning (SF), role–emotional (RE) and mental health (MHI). The SF-12 yields the physical component summary (PCS) and mental component summary (MCS), which measure physical and mental HRQoL, respectively. The PCS and MCS scores range from 0–100, with higher scores indicating better HRQoL. The average PCS and MCS scores in the general US population are both equal to 50 [23]. We calculated the PCS and MCS scores according to the SF-12 scoring algorithm proposed by John E Ware in 1995 [25].

### Data collection

First, four accessible hospitals in Anhui Province were selected. After obtaining verbal permission from the nursing department leaders, the investigator delivered online questionnaires or paper questionnaires to pregnant women in the maternity clinic or ward. An informed consent document was presented on the first page of the questionnaires. Participants who completed the questionnaires were given 5 RMB for their cooperation. The questionnaires took approximately 15 min to complete. The data were collected from October to December 2021, during which time only one family member was allowed to accompany each patient because of the need to prevent and control coronavirus disease 2019 (COVID-19). Most pregnant women were accompanied by female elders or sisters; when a patient’s spouse was present, the investigator asked the spouse to step away as a patient completed the questionnaire to address potential sources of bias. In addition, the investigator who collected the forms by online questionnaires could not see the responses, and each participant was unable to see the responses of the other participants. To ensure clarity and accuracy, investigators were available to answer any questions the participants had regarding the questionnaire items. To collect information on demographic and obstetric characteristics, we used a standardized question: for example, "Do you have a history of adverse pregnancy or childbirth outcomes?".

### Statistical analysis

Data were analysed using SPSS version 25.0 software. We calculated the mean and standard deviation of the participants’ scores for perceived spousal empathy and personal HRQoL. Correlations between the scores for perceived empathy and HRQoL were assessed using the Pearson correlation coefficient.

A *t* test and one-way analysis of variance (ANOVA) were performed to assess how demographic characteristics and obstetric characteristics affected the total QoL, PCS, and MCS scores. Fisher’s least significant difference (LSD) post hoc test was used to determine the group that caused the difference. The influence of perceived empathy on PCS and MCS scores was explored by multiple regression analysis. Prior to the regression analysis, no severe multicollinearity was confirmed according to the tolerance (< 0.10) and the variance inflation factor (< 5). The *P* value was set to < 0.05 (two-tailed test). The normality of the standardized residuals (Shapiro‒Wilk test), homoscedasticity (Durbin–Watson test), and hypothesis of independence (plot) were also confirmed. Age was converted to a discrete numerical variable based on the mean age of the study population.

## Results

Of the 349 responders who were eligible and were invited to participate in the study, 30 declined to participate; the other 319 pregnant women completed questionnaires, some of which had missing values (response rate of 91.4%). The major reasons for declining to participate included physical discomfort or impatience, which occurred mainly in outpatient clinics. Eight questionnaires were excluded because of concerns about their validity because the participants selected the same answer for every question; thus, 311 completed questionnaires were ultimately included.

### Participants' demographic and obstetric characteristics and differences in HRQoL

The mean age of the 311 respondents was 28.7 ± 3.6 years (range, 20–40 years). Most of the participants were educated at or above the college level (*n* = 269, 86.5%), were in the third trimester (*n* = 221, 71.1%) and did not have any pregnancy complications (*n* = 296, 95.2%).

Planned pregnancy, maternal physical condition during pregnancy, and occupational stress had statistically significant effects on the PCS score. Place of residence, educational level, monthly household income per capita, planned pregnancy, pregnancy complications, exercise before pregnancy, adherence to scientific dietary recommendations, sleep, maternal physical condition during pregnancy, and occupational stress had statistically significant effects on the MCS score. The MCS scores of the participants who lived in urban areas were significantly higher than those of participants who lived in rural areas (t = -2.618, *p* = 0.01). Participants with a monthly household income per capita greater than 3000 had higher MCS scores than did those with an income of 3000 or less (t = -2.125, *p* = 0.03). Compared with unplanned pregnancies, planned pregnancies were associated with higher MCS scores (t = -2.427, *p* = 0.02). Adherence to evidence-based dietary recommendations was also linked to higher MCS scores (t = -4.078, *p* < 0.01). Conversely, the presence of pregnancy complications was associated with lower MCS scores (t = 2.358, *p* = 0.02). Post hoc comparisons indicated that participants who exercised more than 5 h per week before pregnancy had significantly higher MCS scores than those who exercised less than 2 h per week before pregnancy (*p* = 0.01). Participants with poor sleep quality had lower MCS scores than those with ordinary sleep (*p* = 0.01) and good sleep quality (*p* < 0.01). Participants in poor physical condition during pregnancy had lower MCS scores than did those in good condition (*p* = 0.03), and those with a ordinary condition had lower scores than did those in good condition (*p* < 0.01). Regarding occupational stress, participants with high stress had lower MCS scores than did those with ordinary stress (*p* = 0.01) and low or no stress (*p* < 0.01), while those with ordinary stress had lower scores than did those with low or no stress (*p* = 0.03). More information is shown in Table 1.

**Table 1:** Demographic and obstetric characteristics of the pregnant women and the differences in the PCS and MCS scores (*n* = 311)

Characteristics	n (%)	PCS score		MCS score	
		Mean (SD)	*P* value	Mean (SD)	*P* value
1. Age(years)			0.79		0.43^a^
< 29	185 (59.5%)	41.5 ± 7.5		48.0 ± 9.3	
≥ 29	126 (40.5%)	41.7 ± 7.9		47.2 ± 8.9	
2. Place of residence			0.31		0.01
Rural area	36 (11.6%)	42.5 ± 8.3		44.0 ± 10.5	
Urban area	275 (88.4%)	41.5 ± 7.6		48.2 ± 8.8	
3. Educational level			0.36		0.02
Junior high school or below	16 (5.1%)	44.7 ± 9.2		44.9 ± 13.6	
Senior high school	26 (8.4%)	42.4 ± 7.9		42.8 ± 9.2	
College	105 (33.8%)	41.4 ± 6.9		48.3 ± 8.7	
Undergraduate or above	164 (52.7%)	41.3 ± 7.9		48.4 ± 8.6	
4. Occupation			0.98		0.09
None	62 (19.9%)	41.7 ± 7.4		45.5 ± 10.0	
Knowledge worker	188 (60.5%)	41.6 ± 7.9		48.4 ± 8.5	
Physical labourer	61 (19.6%)	41.4 ± 7.2		47.7 ± 9.7	
5. Family’s monthly income per capita			0.40		0.03
≤ 3000	36 (11.6%)	42.6 ± 7.7		44.7 ± 9.9	
> 3000	275 (88.4%)	41.5 ± 7.6		48.1 ± 9.0	
6.Height			0.14		0.89
< 162 cm	185 (59.5%)	41.1 ± 7.5		47.8 ± 9.3	
≥ 162	126 (40.5%)	42.4 ± 7.8		47.6 ± 8.9	
7. Pre-pregnancy weight			0.97		0.77
< 50	75 (24.1%)	41.7 ± 7.4		47.5 ± 8.5	
50–60	183 (58.8%)	41.5 ± 7.7		48.0 ± 9.1	
> 60	53 (17.0%)	41.8 ± 8.0		47.0 ± 10.0	
8. Gestational age			0.72		0.81
14–27 weeks	90 (28.9%)	41.4 ± 7.2		47.5 ± 9.3	
≥ 28 weeks	221 (71.1%)	41.7 ± 7.8		47.8 ± 9.1	
9. Gravidity			0.24		0.48
1	219 (70.4%)	41.3 ± 7.4		47.9 ± 8.9	
≥ 2	92 (29.6%)	42.4 ± 8.1		47.1 ± 9.6	
10. Planned pregnancy			0.03		0.02
No	66 (21.2%)	43.5 ± 7.4		45.3 ± 10.0	
Yes	245 (78.8%)	41.1 ± 7.6		48.3 ± 8.8	
11. Use of assisted reproductive technology			0.10		0.75
No	214 (68.8%)	42.1 ± 8.0		47.8 ± 9.5	
Yes	97 (31.2%)	40.6 ± 6.8		47.4 ± 8.4	
12. Parity			0.14		0.22
0	200 (64.3%)	41.1 ± 7.4		48.2 ± 8.8	
≥ 1	111 (35.7%)	42.5 ± 7.9		46.8 ± 9.6	
13. History of adverse pregnancy or childbirth outcomes			0.12		0.12
No	272 (87.5%)	41.9 ± 7.8		48.0 ± 9.2	
Yes	39 (12.5%)	39.8 ± 6.2		45.6 ± 8.3	
14. Pregnancy complications			0.66		0.02
No	296 (95.2%)	41.6 ± 7.6		48.0 ± 9.1	
Yes	15 (4.8%)	40.7 ± 7.6		42.3 ± 7.2	
15. History of smoking and drinking before pregnancy			0.25		0.50
None	301 (96.8%)	41.5 ± 7.7		47.6 ± 9.2	
Smoking and/or alcohol consumption	10 (3.2%)	43.4 ± 4.7		49.6 ± 5.5	
16. Exercise before pregnancy			0.31		0.05
< 2 h per week	138 (44.4%)	42.3 ± 8.1		46.6 ± 9.7	
2 ~ 5 h per week	117 (37.6%)	41.1 ± 6.9		47.8 ± 8.8	
> 5 h per week	56 (18.0%)	40.8 ± 8.0		50.2 ± 7.9	
17. Adherence to scientific dietary recommendations			0.47		< 0.01
No	57 (18.3%)	40.9 ± 7.4		43.3 ± 10.2	
Yes	254 (81.7%)	41.8 ± 7.7		48.7 ± 8.6	
18. Sleep			0.08		< 0.01
Poor	20 (6.4%)	43.6 ± 8.7		40.3 ± 9.4	
Ordinary	186 (59.8%)	40.8 ± 7.3		47.7 ± 9.2	
Good	105 (33.8%)	42.6 ± 7.9		49.1 ± 8.3	
19. Maternal physical condition during pregnancy			< 0.01		< 0.01
Poor	5 (1.6%)	36.2 ± 14.4		41.2 ± 7.6	
Ordinary	171 (55.0%)	39.8 ± 7.4		46.0 ± 9.6	
Good	135 (43.4%)	44.1 ± 6.9		50.0 ± 8.0	
20. Occupational stress			0.01		< 0.01
High	45 (14.5%)	39.6 ± 7.2		42.9 ± 8.9	
Ordinary	140 (45.0%)	40.9 ± 7.1		47.4 ± 8.4	
Low or none	126 (40.5%)	43.1 ± 8.1		49.7 ± 9.4	

*Abbreviations*: *PCS* physical component summary, *MCS* mental component summary, *SD* standard deviation

### Mean scores for perceived empathy and HRQoL

The mean total score of empathy as measured by the IRI in the study was 41.6 (SD 9.0). The dimension with the highest average score for empathy was empathic concern, with a mean score of 2.7 (SD 0.6), while the dimension with the lowest score was personal distress, with a mean score of 1.1 (SD 0.9). On the purpose-designed empathy questionnaire, respondents reported higher behavioural empathy (item 6) and cognitive empathy (item 4), with mean scores of 2.7 (SD 0.8) and 2.7 (SD 0.7), respectively, and lower emotional empathy (item 2), with a mean score of 2.5 (SD 0.8). The mean PCS and MCS scores for pregnant Chinese women were 41.6 (SD 7.6) and 47.7 (SD 9.1), respectively. More information is shown in Table 2.

**Table 2:** Mean scores for pregnant women’s perceived empathy, PCS and MCS (*n* = 311)

Scales	Mean ± SD	Minimum score	Maximum score	Range
IRI total score	41.6 ± 9.0	13.0	69.0	0.0 ~ 88.0
Perspective taking	9.4 ± 1.1	0.0	20.0	0.0 ~ 20.0
Fantasy	10.6 ± 2.9	2.0	21.0	0.0 ~ 24.0
Empathic concern	15.9 ± 3.6	3.0	24.0	0.0 ~ 24.0
Personal distress	5.7 ± 0.4	0.0	20.0	0.0 ~ 20.0
Purpose-designed empathy questionnaire				
Purpose-designed Item 1	3.3 ± 0.8	1.0	5.0	1.0 ~ 5.0
Purpose-designed Item 2	2.5 ± 0.8	1.0	4.0	1.0 ~ 5.0
Purpose-designed Item 3	3.5 ± 0.8	1.0	5.0	1.0 ~ 5.0
Purpose-designed Item 4	2.7 ± 0.7	1.0	4.0	1.0 ~ 5.0
Purpose-designed Item 5	3.6 ± 0.8	1.0	5.0	1.0 ~ 5.0
Purpose-designed Item 6	2.7 ± 0.8	1.0	4.0	1.0 ~ 5.0
SF-12				
Physical component summary (PCS)	41.6 ± 7.6	18.6	58.4	0.0 ~ 100.0
Mental component summary (MCS)	47.7 ± 9.1	21.7	64.2	0.0 ~ 100.0

Direction: Higher scores = better outcomes

*Abbreviations*: *IRI* Interpersonal Reactivity Index; Purpose-designed Item 1: “During pregnancy, my husband is able to deeply resonate with my feelings.” and “How often does this happen?”; Purpose-designed Item 2: “During pregnancy, my husband is able to deeply resonate with my feelings.”; Purpose-designed Item 3: “During pregnancy, my husband understands me objectively.” and “How often does this happen?”; Purpose-designed Item 4: “During pregnancy, my husband understands me objectively.”; Purpose-designed Item 5: “During pregnancy, my husband expresses his understanding to me through verbal or non-verbal communication.” “How often does this happen?”; Purpose-designed Item 6: “During pregnancy, my husband expresses his understanding to me through verbal or non‐verbal communication.”, *SF-12* Medical Outcomes Study 12-item Short-Form Health Survey, *SD* standard deviation

### Correlations between perceived empathy and HRQoL

The scores for all items on the purpose-designed empathy questionnaire were found to be positively correlated with the MCS score, with Pearson correlation coefficients ranging from 0.221 to 0.257 (*P* ≤ 0.01), but not the PCS score (*P* > 0.05), as detailed in Table 3. For the IRI, higher perspective‐taking scores were significantly associated with higher PCS and MCS scores (*P* = 0.02, *P* = 0.03). Higher empathic concern scores were significantly associated with higher MCS scores (*P* < 0.01) and lower PCS scores (*P* = 0.04).

**Table 3:** Correlations of perceived empathy with PCS and MCS scores among pregnant women (*n* = 311)

Variable	1	2	3	4	5	6	7	8	9	10	11	12	13
[1] Perspective taking	1	0.408^a^	0.132^b^	0.335^a^	0.815^a^	0.259^a^	0.415^a^	0.361^a^	0.461^a^	0.373^a^	0.402^a^	0.128^b^	0.125^b^
[2] Fantasy		1	0.046	0.337^a^	0.712^a^	0.204^a^	0.176^a^	0.200^a^	0.144^b^	0.091	0.058	0.025	0.016
[3] Empathic concern			1	-0.453^a^	0.255^a^	0.371^a^	0.207^a^	0.384^a^	0.288^a^	0.330^a^	0.267^a^	-0.118^b^	0.229^a^
[4] Personal distress				1	0.575^a^	-0.097	-0.001	-0.093	-0.031	-0.058	-0.066	0.068	-0.154^a^
[5] IRI total score					1	0.290^a^	0.333^a^	0.342^a^	0.361^a^	0.306^a^	0.279^a^	0.053	0.079
[6]Purpose-designed item 1						1	0.523^a^	0.713^a^	0.464^a^	0.594^a^	0.498^a^	-0.005	0.224^a^
[7] Purpose-designed item 2							1	0.562^a^	0.742^a^	0.529^a^	0.681^a^	0.1	0.248^a^
[8] Purpose-designed item 3								1	0.631^a^	0.632^a^	0.583^a^	0.054	0.221^a^
[9] Purpose-designed item 4									1	0.647^a^	0.783^a^	0.074	0.243^a^
[10] Purpose-designed item 5										1	0.664^a^	0.051	0.221^a^
[11] Purpose-designed item 6											1	0.099	0.257^a^
[12] PCS												1	0.042
[13] MCS													1

*Abbreviations*: *IRI* Interpersonal Reactivity Index; purpose-designed items 1 to 6 are the same as purpose-designed items 1 to 6 in Table 2, *MCS* mental component summary, *PCS* physical component summary

^a^Correlation is significant at the 0.01 level (two-tailed). ^b^Correlations are significant at the 0.05 level (two-tailed)

Notably, the scores for all items on the purpose-designed empathy questionnaire were positively linked to the perspective‐taking score (ranging from 0.259 to 0.461, *P* < 0.01), empathic concern score (ranging from 0.207 to 0.371, *P* < 0.01), and total IRI score (ranging from 0.279 ~ 0.361, *P* < 0.01) but not to the personal distress score (*P* > 0.05). Higher scores on item 1, item 2, item 3, and item 4 of the purpose-designed empathy questionnaires were significantly associated with higher fantasy scores (ranging from 0.144 to 0.204, *P* < 0.01), while the same was not true for item 5 and item 6 (*P* > 0.05).

### Multiple linear stepwise regression of HRQoL

Multiple linear regression analysis was used to establish a linear regression model, including variables that were statistically significant for empathy and HRQoL in the correlation analysis and the variables with statistical significance in the single-factor analysis. The results showed that the variance inflation factors of the two models' independent variables were all < 5, indicating that there were no serious multicollinearity problems between the variables [26].

First, the PCS score was treated as the dependent variable, while perspective taking, empathic concern measured by the IRI, planned pregnancy, maternal physical condition during pregnancy and occupational stress were the independent variables (Table 4). The results of the model showed that demographic variables could explain 12.3% of the variation in PCS (*F* = 14.302, *P* < 0.01, *R*^2^ = 0.123). Maternal physical condition during pregnancy, planned pregnancy, and occupational stress were predictors of the PCS score (β = 0.281, *P* < 0.01; β = 0.132, *P* = 0.02; β = -0.128, *P* = 0.02). However, neither perspective taking nor empathic concern was recognized as a statistically significant predictor of the PCS score.

**Table 4:** Multivariate linear regression analysis of the factors associated with the PCS score among pregnant women (*n* = 311)

Model	Standardized coefficients	*t*	*p*	B(95% CI)	F	*P*
	Beta					
(Constant)		11.560	< 0.01	27.146 ~ 38.284	14.302	< 0.01
Maternal physical condition during pregnancy	0.281	5.216	< 0.01	2.542 ~ 5.623		
Occupational stress	0.132	2.449	0.02	0.285 ~ 2.613		
Planned pregnancy	-0.128	-2.394	0.02	-4.343 ~ 0.425		

*R*^2^ = 0.123, Adj. *R*^2^ = 0.114. Maternal physical condition during pregnancy (1 = poor, 2 = ordinary, 3 = good); occupational stress (1 = high, 2 = ordinary, 3 = low or none); and planned pregnancy (1 = no, 2 = yes)

The direction is indicated by the sign of the standardized coefficient: positive coefficients ( +) indicate a positive relationship (better outcomes with higher scores), and negative coefficients (-) indicate a negative relationship (worse outcomes with higher scores)

*Abbreviation*: *PCS* physical component summary

Second, the MCS score was treated as the dependent variable, and the empathy scores measured by six items of the purpose-designed empathy questionnaire were included as independent variables, along with perspective taking, empathic concern, and personal distress. In addition, the statistically significant variables (place of residence, educational level, monthly income per capita, planned pregnancy, pregnancy complications, exercise before pregnancy, adherence to scientific dietary recommendations, sleep, maternal physical condition during pregnancy, and occupational stress) were also treated as independent variables. Item 6 of the purpose-designed empathy questionnaire, occupational stress, adherence to scientific dietary recommendations, educational level, maternal physical condition during pregnancy, planned pregnancy, and empathic concern were recognized as statistically significant predictors of the MCS score. As shown in Table 5, the model explained 22.0% of the variance in pregnant women's MCS scores (*F* = 12.228, *P* < 0.01). Item 6 of the purpose-designed empathy questionnaire and empathic concern were important predictors of the MCS score (β = 0.127, *P* = 0.02; β = 0.158, *P* < 0.01).

**Table 5:** Multivariate linear regression analysis of the factors associated with the MCS score among pregnant women (*n* = 311)

Model	Standardized coefficients	*t*	*p*	B(95% CI)	F	*P*
	Beta					
(Constant)		2.238	0.03	1.170 ~ 18.200	12.228	< 0.01
Purpose-designed item 6	0.127	2.298	0.02	0.221 ~ 2.862		
Occupational stress	0.220	4.155	< 0.01	1.518 ~ 4.248		
Adherence to scientific dietary recommendations	0.127	2.421	0.03	0.561 ~ 5.432		
Educational level	0.120	2.243	0.03	0.161 ~ 2.460		
Maternal physical condition during pregnancy	0.167	3.085	< 0.01	1.050 ~ 4.750		
Empathic concern	0.158	2.875	< 0.01	0.126 ~ 0.671		
Planned pregnancy	0.105	2.061	0.04	0.106 ~ 4.591		

*R*^2^ = 0.220, Adj. *R*^2^ = 0.202

Purpose-designed item 6 (1 = poor, 2 = ordinary, 3 = good and 4 = very good); occupational stress (1 = high, 2 = ordinary, 3 = low or none); adherence to scientific dietary recommendations (1 = no, 2 = yes); educational level (1 = junior high school or below, 2 = senior high school, 3 = college, 4 = undergraduate or above); maternal physical condition during pregnancy (1 = poor, 2 = ordinary, 3 = good); and planned pregnancy (1 = no, 2 = yes)

The direction is indicated by the sign of the standardized coefficient: positive coefficients ( +) indicate a positive relationship (better outcomes with higher scores)

*Abbreviations*: Purpose-designed item 6: “During pregnancy, my husband expresses his understanding to me through verbal or non‐verbal communication”, which reflects behavioural empathy, *MCS* mental component summary

## Discussion

### Research purpose and innovation

The present study attempted to examine pregnant women's perceived empathy from their spouses and whether perceived empathy plays a role in HRQoL among pregnant women in mainland China. This was among the first studies on pregnant women's perceptions of different components of empathy and the relationship between these components and HRQoL in the nursing field. The findings of the present study may provide insights for nursing leaders and nursing educators in managing pregnant women’s perceived empathy and HRQoL.

### Pregnant women perceived lower empathy from their spouses

The mean score on the perceived IRI-C from spouses among Chinese pregnant women in our study was slightly lower than values reported in previous studies focusing on empathy. Due to the lack of previous research examining perceived empathy from the perspective of recipients, direct comparison of the findings of the present study with those of other studies is challenging. Additionally, perceived empathy was lower than that reported by previous related empathy studies, which applied the Chinese version of the IRI to assess individual empathy for others among 281 young breast cancer patients and their spouses (68.05 for the breast cancer patients and 62.27 for their spouses) [17] and individual levels of empathy among 168 couples experiencing infertility (46.85 for the wives and 47.90 for their husbands) in China [18]. The reason for this difference may be that the empathy expressed by the husbands may not be fully perceived by the wives, resulting in differences [18]. This indicates that the level of empathy perceived by pregnant women may be lower than the actual level of empathy given by their spouses. In addition, spouses may have lower levels of empathy with their partners during pregnancy than during severe gynaecological diseases and infertility, even though pregnancy is an important stage of life.

### Correlation analysis between the purpose-designed empathy questionnaire and the IRI

The current study used both the IRI and a purpose-designed questionnaire to evaluate the status of perceived empathy in pregnant Chinese women. The purpose-designed questionnaire was used to measure the level and frequency of perceived emotional empathy, cognitive empathy, and behavioural empathy. Correlation analysis showed that the six items of the purpose-designed empathy questionnaire were significantly positively correlated with perspective taking and empathic concern but were not correlated with the dimension of personal distress and were only partially correlated with the dimension of fantasy. This result incidentally confirms that the IRI may indeed measure variables unrelated to empathy, including personal distress and fantasy, in line with the findings of scholars such as Baron‐Cohen [27]. Future research should focus on the scientific nature of the IRI, a widely used empathy measurement tool, and develop an empathy scale that assesses emotional empathy, cognitive empathy, and behavioural empathy.

### The PCS and MCS scores of pregnant women were below the general averages

The PCS (41.6 ± 7.6) and MCS (47.7 ± 9.1) scores were below the average of 50 in the general US population [23]. Moreover, they were below the averages for the urban population of Chengdu (51.2 ± 6.6; 49.9 ± 7.7) [28], the population of Hong Kong (50.2 ± 7.0; 48.4 ± 8.8) [24] and the urban population of Australia (49.3 ± 9.9; 52.0 ± 8.8) [29]. Moreover, some of the participants were recruited from wards; hospitalized pregnant women are in poorer health than the general population. Therefore, appropriate interventions for pregnant women are needed to promote high HRQoL scores.

### Multiple regression analysis of PCS and MCS scores

The main statistically significant finding of this study was that item 6 of the purpose-designed empathy questionnaire, which assesses the behavioural empathy component, was a positive predictor of the MCS score. Hence, pregnant women with greater perceived behavioural empathy from their spouses had better mental health. The results verified that pregnant women’s perceived behavioural empathy had positive effects on them, indicating that it is important for spouses to show concern and understanding towards pregnant women through verbal and nonverbal communication. However, there is no empirical research on behavioural empathy in nursing fields. Additionally, empathic concern was recognized as a significant predictor of MCS scores. Pregnant women with greater perceived empathic concern had better mental health.

These results are similar to those of a study on healthy couples by Rosen et al. [30]. That study showed that the perception of empathy from one’s spouse increases one’s sense of marital intimacy [30] and thus one’s HRQoL. The reason is that when a pregnant woman perceives greater behavioural empathy and empathic concern from her spouse–caregiver, such in-depth understanding and psychological resonance from her spouse, she can establish an interdependent relationship with her spouse at the emotional, cognitive, and behavioural levels, thus improving her HRQoL, especially the mental component. In contrast, a pregnant woman's perceived low empathy from her spouse may be misinterpreted as neglect or a lack of concern for her inner pain [17], which can lead to emotional dissatisfaction and a lower MCS score. Therefore, hospital managers, nursing educators, and nursing researchers should emphasize family members' behavioural empathy towards pregnant women.

This study revealed that perceived emotional empathy, cognitive empathy, and behavioural empathy were not predictors of PCS in pregnant women. This finding is inconsistent with findings regarding patients’ perceived empathy from their physicians, which contributes to patients' physical and psychological quality of life [31]. These differences may be attributable to differences in the research participants, measurement tools for perceived empathy, and quality of life. Moreover, correlation analysis revealed that empathic concern, as measured by the IRI, was negatively correlated with the PCS score (R = -0.118, *P* = 0.04). In Chinese culture, the spouse is the woman's closest and most trusted partner, and most women are more willing to display vulnerability when under a spouse's care, especially during pregnancy. A spouse's perceived empathic concern may cause a feeling of self-pity in a pregnant woman and amplify her physical discomfort. Additionally, relatively speaking, the emotional state of an individual changes faster than the physiological state; thus, the perceived empathy given by the spouse may greatly improve the emotional state in the short term, but the physical state may take a longer time to change. Further multicentre, longitudinal, and comparative studies are warranted to examine the role of pregnant women’s perceptions of cognitive empathy, emotional empathy, and behavioural empathy in relation to their PCS and MCS scores.

### Strengths

Our study uniquely contributed to the literature by examining perceived empathy from a recipient’s perspective within the family context, particularly during pregnancy, which is a critical period for emotional support. Utilizing a self-designed questionnaire, despite its limitations, we explored cognitive, emotional, and behavioural empathy, offering a comprehensive view that enriches our understanding and paves the way for future research. The findings revealed the relationships between different types of perceived empathy, especially behavioural empathy, and PCS and MCS scores in a cohort of pregnant Chinese women, providing valuable insights for healthcare professionals and researchers interested in the role of empathy in health outcomes and well-being.

### Limitations

First, this study adopted a cross-sectional design, which limits the possibility of establishing causal relationships between perceived empathy and HRQoL. Longitudinal and experimental studies are warranted to clarify the causal relationship between perceived empathy and HRQoL among pregnant Chinese women to support an in-depth understanding of the dynamic development and mechanism of correlation between empathy (cognitive, emotional, and behavioural empathy) and HRQoL (PCS and MCS scores). Second, behavioural empathy was measured by only one simple purpose-designed item; the observations would be more rigorous and scientific if additional scales were used for confirmation. In addition, the lack of studies on empathy from spouses, particularly behaviour empathy, makes it difficult to conduct a deep and adequate discussion, although this is one of the innovative aspects of this research. Finally, the study was conducted during the period of standard prevention and control measures for COVID-19, during which pregnant women had restricted daily routines and social interactions; limited access to education/information; and more complicated routine prenatal care, hospitalization, and family care regulations, which produced negative emotional effects on these women, including anxiety, frustration, fear, depression, and concerns about their own health and that of their children [32, 33].

## Conclusion

Perceived empathy from spouses and HRQoL were lower for pregnant women in the second and third trimesters of pregnancy. Nursing managers and pregnant women should raise awareness regarding the concern of perceived empathy from spouses. Additional studies further exploring this aspect are therefore necessary. Moreover, future research could also focus on specific subsets of pregnant women, such as those with high-risk pregnancies, and explore the role of spousal empathy in their physical and mental state, considering that pregnant women may need more spousal empathy than pregnant women in general.

## Data Availability

The data of this study are available from the corresponding author upon request.

## Abbreviations

HRQoL: Health-related quality of life
IRI: Interpersonal Reactivity Index
SF-12: The Medical Outcomes Study 12-item Short-Form Health Survey
MCS: Mental component summary
PCS: Physical component summary
